# Luseogliflozin restores GIP responsiveness in diabetic male mice with preserved β‐cell function

**DOI:** 10.14814/phy2.71016

**Published:** 2026-07-11

**Authors:** Quan Yingyue, Harumi Takahashi, Norihide Yokoi, Wataru Ogawa, Kenji Sugawara

**Affiliations:** ^1^ Division of Diabetes and Endocrinology, Department of Internal Medicine Kobe University Graduate School of Medicine Kobe Japan; ^2^ Division of Molecular and Metabolic Medicine Kobe University Graduate School of Medicine Kobe Japan; ^3^ Laboratory of Animal Breeding and Genetics, Division of Applied Biosciences, Graduate School of Agriculture Kyoto University Kyoto Japan; ^4^ Division of Metabolic Diseases, Department of Translational Medical Science Kobe University Graduate School of Medicine Kobe Japan

**Keywords:** diabetes, incretin, insulin secretion, luseogliflozin

## Abstract

Impaired glucose‐dependent insulinotropic polypeptide (GIP)‐induced insulin secretion is a characteristic feature of type 2 diabetes, whereas glucagon‐like peptide‐1 (GLP‐1) responsiveness is relatively preserved. Our previous study proposed that chronic β‐cell depolarization alters G protein signaling and contributes to this differential incretin responsiveness, but whether glucose lowering by sodium–glucose cotransporter 2 inhibition restores incretin responsiveness remains unclear. We investigated the effects of chronic luseogliflozin treatment on incretin responsiveness in two diabetic mouse models, KK‐Ay mice with preserved residual β‐cell function and streptozotocin/high‐fat diet‐induced diabetic mice with severe β‐cell impairment. Luseogliflozin improved glycemic control in both models without marked changes in body weight or plasma insulin levels. In KK‐Ay mice, luseogliflozin restored the glucose‐lowering effect of GIP during GIP‐preload glucose tolerance test. In contrast, this effect was not observed in streptozotocin/high‐fat diet‐induced diabetic mice, suggesting that preserved residual β‐cell function is required for recovery of GIP responsiveness. Ex vivo analyses using isolated islets further showed that chronic luseogliflozin treatment altered incretin‐stimulated insulin secretion, including attenuation of enhanced GLP‐1 responsiveness under diabetic conditions. These findings suggest that luseogliflozin may partially normalize aberrant β‐cell incretin signaling and restore GIP responsiveness when sufficient β‐cell function remains.

## 1 INTRODUCTION

Glucose homeostasis is maintained through the regulation of insulin secretion from pancreatic β‐cells in response to various physiological stimuli. Among these factors, glucose is the primary trigger of insulin secretion (Henquin, 2009; Prentki et al., 2013; Seino et al., 2010), and glucose‐induced insulin secretion (GIIS) is mediated by a series of processes initiated by glucose metabolism within β‐cells. In addition, GIIS is further potentiated by hormones and neurotransmitters. Many of these factors exert their effects via activation of the heterotrimeric G proteins Gs and Gq, which signal through the cyclic AMP (cAMP) and diacylglycerol (DAG)/inositol 1,4,5‐trisphosphate (IP₃) pathways, respectively (Ahrén, 2009; Prentki & Matschinsky, 1987; Seino & Shibasaki, 2005). Among them, the incretin hormones glucagon‐like peptide‐1 (GLP‐1) and glucose‐dependent insulinotropic polypeptide (GIP) are secreted from intestinal L cells and K cells after meal ingestion and play crucial roles in preventing postprandial hyperglycemia by enhancing insulin secretion in a glucose‐dependent manner (Baggio & Drucker, 2007; Drucker, 2006; Holst, 2007; Yabe & Seino, 2011).

However, in type 2 diabetes (T2D), GIP‐induced insulin secretion (GIP‐IIS) is markedly impaired, whereas GLP‐1‐induced insulin secretion (GLP‐1‐IIS) is relatively preserved, which explains the unique therapeutic efficacy of GLP‐1–based therapies (Drucker & Nauck, 2006; Elahi et al., 1994; Holst & Gromada, 2004; Nauck et al., 1993; Yamada et al., 2006). Animal studies suggest that impaired GIP‐induced insulin secretion in diabetes is due to reduced GIP receptor expression in β‐cells. In contrast, GLP‐1 retains its insulinotropic effect despite reported reductions in GLP‐1 receptor expression (Lynn et al., 2001; Lynn et al., 2003; Rajan et al., 2015; Xu et al., 2007; Younan & Rashed, 2007). This concept is consistent with the Gs/Gq signaling switch hypothesis proposed in a previous study from our group, which provides a mechanistic explanation for the differential incretin responses observed in diabetes (Oduori et al., 2020). In that study, sustained depolarization of pancreatic β‐cells was shown to shift the dominant signaling pathway mediating insulin secretion from Gs‐dependent to Gq‐dependent signaling (Oduori et al., 2020).

Luseogliflozin is a selective sodium–glucose cotransporter 2 (SGLT2) inhibitor widely used for the treatment of T2D (Seino et al., 2018; Uchida et al., 2015). Although its systemic glucose‐lowering effects are well established, its impact on incretin responsiveness and G protein‐mediated signaling dynamics within β‐cells remains largely unclear. In this study, we investigated the effects of chronic luseogliflozin treatment on incretin responsiveness in β‐cells using two diabetic mouse models, KK‐Ay mice representing diabetes with preserved residual β‐cell function and streptozotocin/high‐fat diet (STZ‐HFD) mice representing diabetes with severe β‐cell impairment. Specifically, we aimed to determine whether luseogliflozin treatment restores GIP responsiveness under diabetic conditions and to clarify whether this response depends on preserved residual β‐cell function.

## 2 MATERIALS AND METHODS

### 2.1 Animals and experimental design

Male KK‐Ay/TaJcl (KK‐Ay) and C57BL/6J mice were purchased from CLEA Japan, Inc. (Tokyo, Japan). Mice were maintained under controlled environmental conditions at 22°C ± 2°C with 55% ± 10% humidity under a 12‐h light/dark cycle, with free access to standard chow (CLEA Rodent Diet CE‐2; CLEA Japan, Inc., Tokyo, Japan) and water. All animal experiments were approved by the Institutional Animal Care and Use Committee of Kobe University Graduate School of Medicine (approval no. 2021‐08‐03) and were conducted in accordance with institutional and national guidelines for the care and use of laboratory animals. Mice were anesthetized with isoflurane (Fujifilm Wako, Osaka, Japan; catalog no. 099‐06571) and euthanized by cervical dislocation before pancreatic islet isolation.

Ten‐ to twelve‐week‐old male KK‐Ay mice were randomly assigned to control or luseogliflozin‐treated groups. Luseogliflozin was administered by feeding chow containing 0.01% luseogliflozin (Taisho Pharmaceutical Co., Ltd., Tokyo, Japan) for 5 weeks. Control mice received standard chow. Body weight and random blood glucose levels were monitored during the treatment period to evaluate the metabolic effect of chronic SGLT2 inhibition. For the streptozotocin/high‐fat diet‐induced diabetic mouse model, 8‐week‐old male C57BL/6J mice were fed a high‐fat diet containing approximately 60% kcal from fat (High Fat Diet 32 [HFD32], CLEA Japan, Inc.) and then received intraperitoneal injections of streptozotocin (STZ; Sigma‐Aldrich, St. Louis, MO, USA; catalog no. S0130) at 20 mg/kg/day for three consecutive days.

### 2.2 Metabolic measurements

Body weight and random blood glucose levels were measured during the treatment period. Blood glucose levels were determined using an Antsense Duo glucose analyzer (Horiba, Kyoto, Japan). Blood samples were collected from the tail vein. Plasma insulin concentrations were measured using an Ultra‐Sensitive Mouse/Rat Insulin ELISA Kit (Morinaga Institute of Biological Science, Yokohama, Japan; catalog no. M1104) according to the manufacturer's instructions.

### 2.3 GIP‐preload oral glucose tolerance test

After a 6‐h fast, mice received an intraperitoneal preload of either GIP (50 μg/mouse; Peptide Institute, Inc., Osaka, Japan; catalog no. 4178‐V) or vehicle (Milli‐Q water), followed immediately by an oral glucose load (1.5 g/kg body weight; D‐(+)‐glucose, Sigma‐Aldrich; catalog no. G8270). Tail‐vein blood glucose levels were measured at 0, 15, 30, 60, 90, and 120 min after injection. The area under the curve (AUC) for blood glucose was calculated to assess glucose tolerance and incretin responsiveness. The comparison between vehicle‐preload and GIP‐preload conditions was used to evaluate the glucose‐lowering effect of exogenous GIP in vivo.

### 2.4 Islet isolation and insulin secretion assay

Pancreatic islets were isolated from luseogliflozin‐treated and control mice (KK‐Ay and STZ‐HFD models) by collagenase digestion (Collagenase P; Roche Diagnostics, Mannheim, Germany; catalog no. 11249002001), as previously described (Oduori et al., 2020; Yingyue et al., 2023). For insulin secretion assays, size‐matched islets were preincubated for 30 min at 37°C in Krebs–Ringer bicarbonate HEPES buffer containing 2.8 mM glucose. Groups of 5–10 islets per well were then incubated for 30 min in buffer containing 2.8 or 11.1 mM glucose, with or without 1 nM GIP or 1 nM GLP‐1 (Peptide Institute, Inc.; catalog no. 4344‐V). After stimulation, supernatants were collected for measurement of secreted insulin. Intracellular insulin content was extracted by adding KRBH buffer containing 0.1% Triton X‐100. Insulin secretion was expressed as a percentage of total insulin content. Independent islet preparations were used as biological replicates, and the number of independent experiments is indicated in the figure legends.

### 2.5 Statistical analysis

All results are presented as means ± SD unless otherwise stated. The experimental unit was the individual mouse for in vivo studies and the independent islet preparation for ex vivo studies. Sample sizes were determined based on previous studies and experimental feasibility. Comparisons between two groups were made using the unpaired Student's *t*‐test. For multiple‐group comparisons, one‐way ANOVA followed by Tukey's post hoc test was applied. Statistical significance was set at *p* < 0.05. All analyses were performed using GraphPad Prism 10 (GraphPad Software, San Diego, CA, USA).

## 3 RESULTS

### 3.1 Chronic luseogliflozin treatment improves glycemic control and restores GIP responsiveness in KK‐ay mice

**FIGURE 1:**
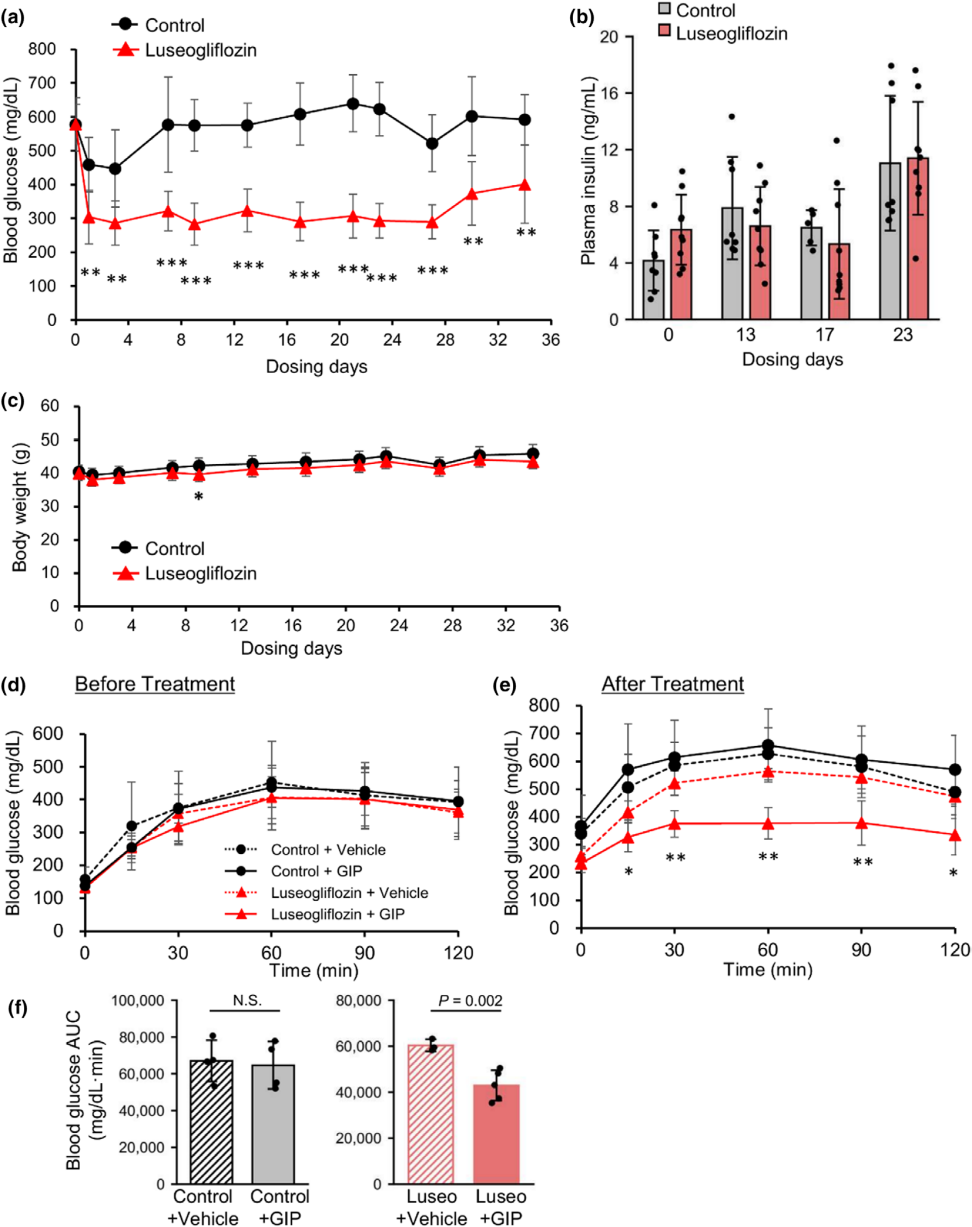
Chronic luseogliflozin treatment restores GIP responsiveness in KK‐Ay mice. KK‐Ay mice were treated with either control diet or luseogliflozin‐containing diet for 5 weeks. (a–c) Random blood glucose levels (a), plasma insulin levels (b), and body weight (c) were monitored during the treatment period. (d, e) GIP‐preload glucose tolerance tests were performed before treatment (d) and after 5 weeks of treatment (e). Vehicle or GIP was administered before oral glucose loading, and blood glucose levels were measured at the indicated time points. (f) Quantification of glucose excursion during the GIP‐preload glucose tolerance test after treatment, expressed as the area under the curve (AUC) for blood glucose levels in (e). Data are presented as means ± SD. *n* = 8–9 mice per group. Experiments were performed in three independent cohorts. Statistical analysis was performed using unpaired Student's *t*‐test for two‐group comparisons and one‐way ANOVA followed by Tukey's post hoc test for multiple comparisons. *p < 0.05, **p < 0.01, ***p < 0.001. Luseo, luseogliflozin; N.S., not significant.

Chronic administration of luseogliflozin for 5 weeks significantly reduced random blood glucose levels in KK‐Ay mice from the early phase after treatment (Figure 1a). Random plasma insulin levels were not significantly different between the two groups (Figure 1b). In addition, no significant changes in body weight were observed (Figure 1c).

To evaluate incretin‐mediated insulinotropic effects, a GIP‐preload oral glucose tolerance test (GIP‐preload OGTT) was performed before and after 5 weeks of luseogliflozin treatment. Prior to treatment, KK‐Ay mice showed no significant glucose‐lowering response to GIP (Figure 1d), indicating impaired GIP responsiveness under diabetic conditions.

In contrast, after 5 weeks of treatment, vehicle‐treated mice still exhibited no response to GIP, whereas luseogliflozin‐treated mice showed a marked restoration of GIP responsiveness, with significant improvements in both glucose excursion curves and area under the curve (AUC) (Figure 1e,f). Taken together, these results demonstrate that luseogliflozin improves impaired GIP responsiveness in KK‐Ay mice.

### 3.2 GIP responsiveness is not restored in STZ‐HFD mice

**FIGURE 2:**
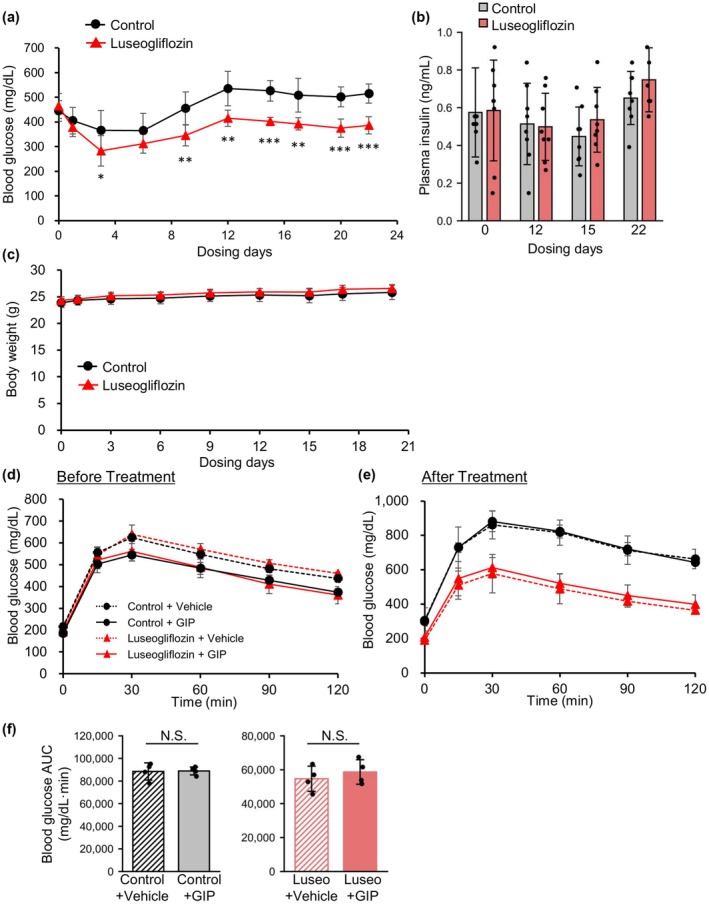
Luseogliflozin does not restore GIP responsiveness in STZ‐HFD mice. STZ‐HFD mice were treated with vehicle or luseogliflozin for 4 weeks. (a–c) Random blood glucose levels (a), plasma insulin levels (b), and body weight (c) were monitored during treatment. (d, e) GIP‐preload glucose tolerance tests were performed before treatment (d) and after luseogliflozin treatment (e). Vehicle or GIP was administered before glucose loading, and blood glucose levels were measured at the indicated time points. After luseogliflozin treatment, GIP preload did not significantly alter glucose excursion compared with vehicle preload. (f) Quantification of glucose excursion during the GIP‐preload glucose tolerance test after treatment, expressed as the area under the curve (AUC) for blood glucose levels in (e). Data are presented as means ± SD. *n* = 8–9 mice per group. Experiments were performed in three independent cohorts. Statistical analysis was performed using unpaired Student's *t*‐test for two‐group comparisons and one‐way ANOVA followed by Tukey's post hoc test for multiple comparisons. *p < 0.05, **p < 0.01, ***p < 0.001. Luseo, luseogliflozin; N.S., not significant.

We next performed similar analyses in STZ‐HFD mice, a model of β‐cell–deficient diabetes. Chronic luseogliflozin treatment significantly reduced random blood glucose levels (Figure 2a), whereas plasma insulin levels were not significantly different between groups (Figure 2b). Body weight was also comparable between the two groups, consistent with the findings in KK‐Ay mice (Figure 2c).

In the GIP‐preload OGTT, a modest but non‐significant improvement in glucose tolerance was observed with GIP administration in mice before treatment (Figure 2d). However, after 4 weeks of luseogliflozin treatment, no improvement in glucose tolerance in response to GIP was observed in either group, and this was confirmed by the AUC analysis (Figure 2e,f). These results indicate that, in conditions of severe β‐cell loss, normalization of blood glucose alone is insufficient to restore GIP responsiveness.

### 3.3 Luseogliflozin alters GLP‐1 responsiveness in islets from diabetic mouse models

To examine changes in incretin responsiveness at the cellular level, we evaluated insulin secretion under GLP‐1 and GIP stimulation using isolated islets from each mouse model.

**FIGURE 3:**
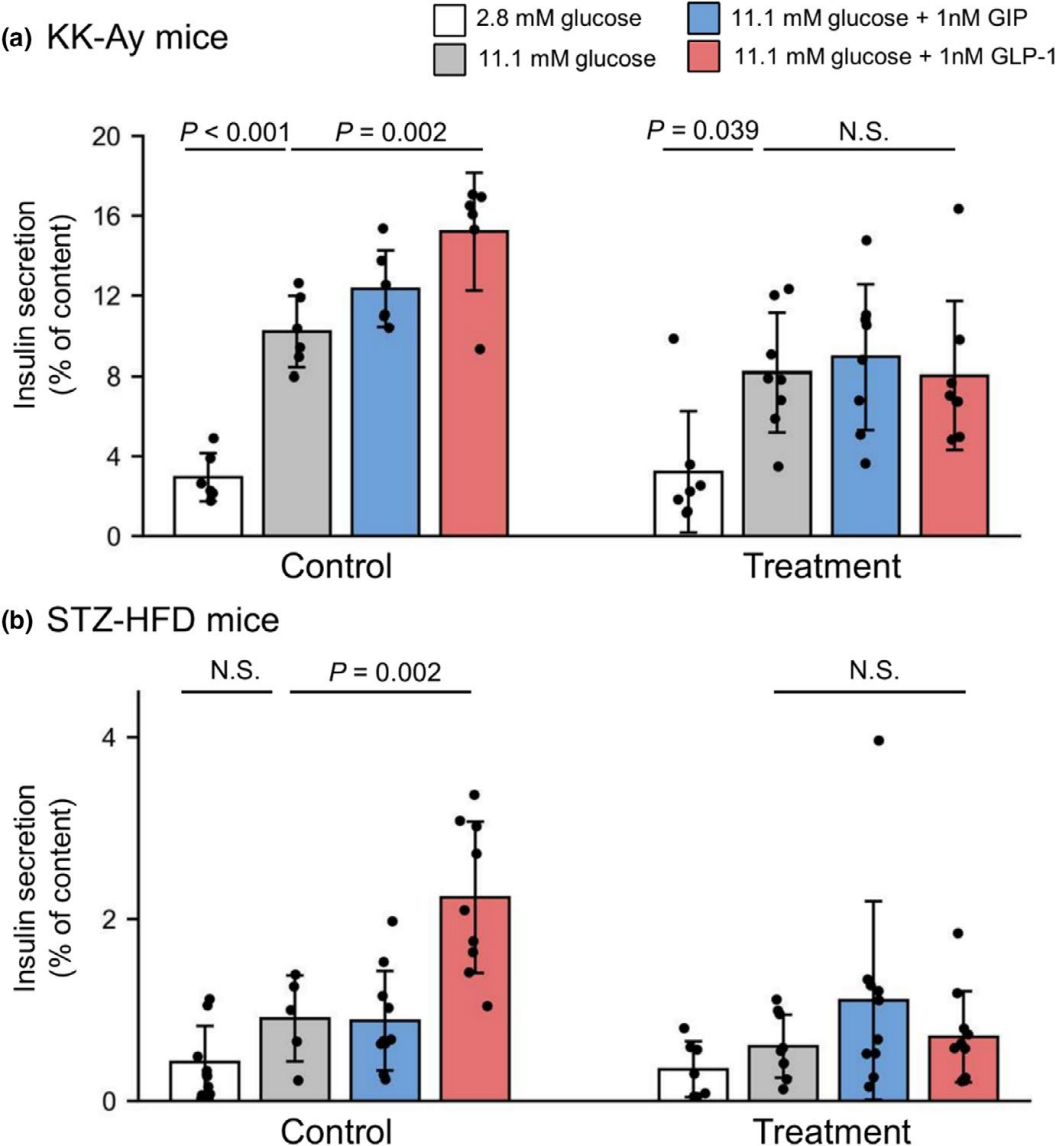
Luseogliflozin alters incretin‐stimulated insulin secretion in isolated islets. Pancreatic islets were isolated from KK‐Ay mice and STZ‐HFD mice after treatment. Isolated islets were incubated under low‐glucose or high‐glucose conditions, with or without incretin stimulation, and insulin secretion was assessed ex vivo. (a) Insulin secretion from isolated KK‐Ay mouse islets stimulated with 2.8 mM glucose, 11.1 mM glucose, 11.1 mM glucose plus 1 nM GIP, or 11.1 mM glucose plus 1 nM GLP‐1. (b) Insulin secretion from isolated STZ‐HFD mouse islets under the same stimulation conditions. Insulin secretion is expressed as a percentage of total insulin content. Data are presented as means ± SD. Experiments were performed using three independent islet preparations. Statistical analysis was performed using one‐way ANOVA followed by Tukey's post hoc test for multiple comparisons. Exact *p* values are indicated in the figure. N.S., not significant.

In islets isolated from KK‐Ay mice, GLP‐1 stimulation under high‐glucose conditions significantly increased insulin secretion in the control group. In contrast, chronic luseogliflozin treatment markedly attenuated the insulinotropic response to GLP‐1 stimulation (Figure 3a).

Similarly, in islets isolated from STZ‐HFD mice, insulin secretion under both low‐ and high‐glucose conditions was generally reduced, whereas GLP‐1 stimulation induced a relatively strong insulin secretory response in the control group. In contrast, this GLP‐1‐induced response was significantly reduced in the luseogliflozin‐treated group (Figure 3b).

Taken together, these findings indicate that chronic luseogliflozin treatment attenuates the enhanced GLP‐1‐dependent insulin secretory response observed in islets from diabetic mouse models.

## 4 DISCUSSION

In the present study, we demonstrated that chronic luseogliflozin treatment improved glycemic control in both KK‐Ay mice and STZ‐HFD diabetic mice without substantially altering body weight or plasma insulin levels. Luseogliflozin restored GIP responsiveness in KK‐Ay mice, whereas no such recovery was observed in STZ‐HFD mice. Furthermore, ex vivo analysis using isolated islets showed that incretin responsiveness was altered in islets from luseogliflozin‐treated mice, with a particular reduction in the enhanced GLP‐1 responsiveness observed under diabetic conditions.

**FIGURE 4:**
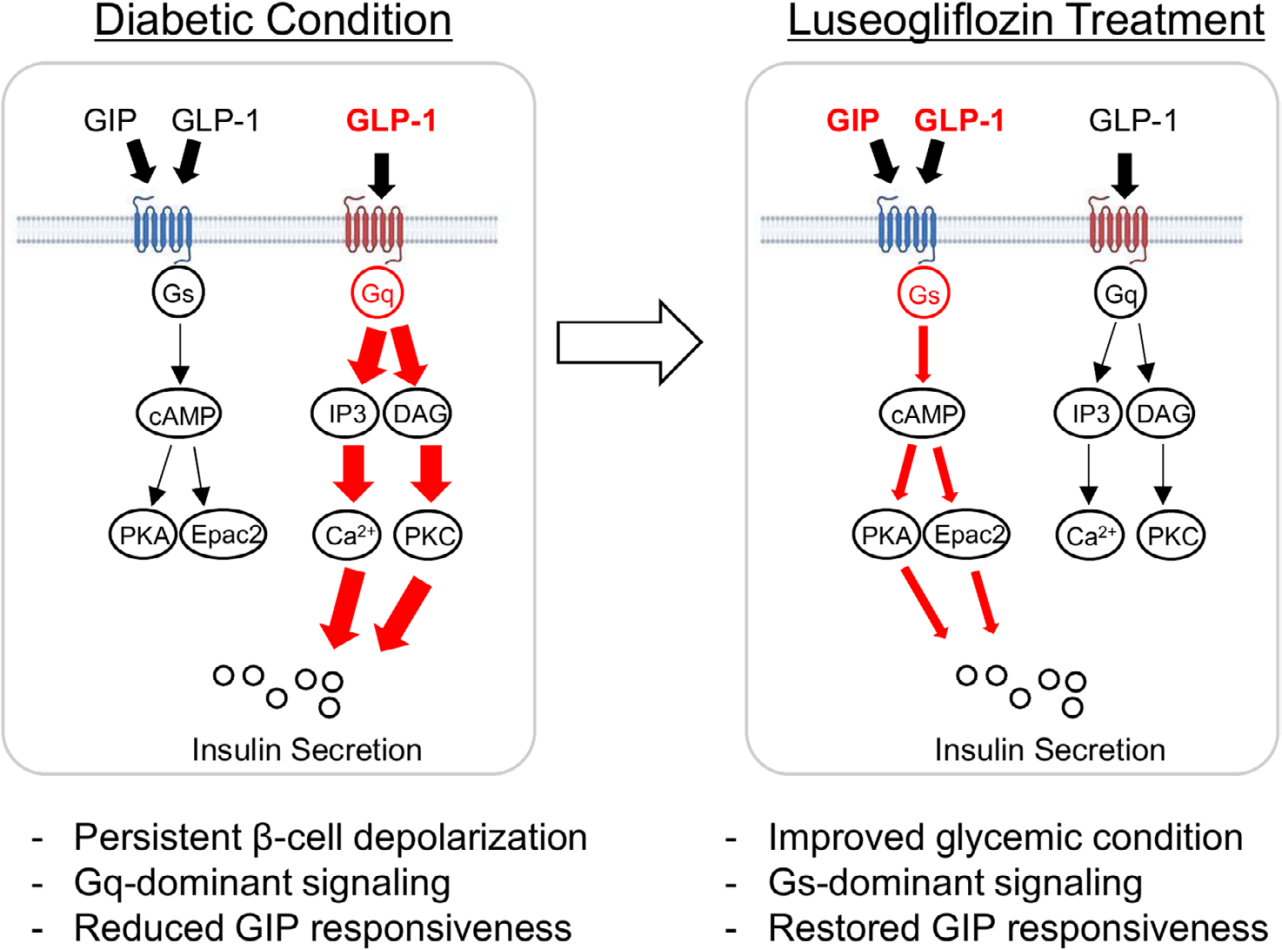
Proposed model for restoration of GIP responsiveness by luseogliflozin. Under diabetic conditions, chronic hyperglycemia and persistent β‐cell depolarization promote a shift from Gs‐dominant to Gq‐dominant incretin signaling. In this state, GLP‐1 can still amplify insulin secretion through Gq‐dependent signaling, whereas GIP responsiveness is reduced because GIP mainly acts through the Gs–cAMP pathway. Luseogliflozin improves hyperglycemia and may alleviate the diabetic β‐cell state, thereby restoring Gs‐dominant incretin signaling and enhancing GIP responsiveness.

Under physiological conditions, incretin hormones primarily amplify glucose‐induced insulin secretion through the Gs–cAMP–PKA/Epac2 pathway (Oduori et al., 2020). GLP‐1 receptor signaling can involve not only Gs but also Gq‐mediated pathways, whereas GIP receptor signaling is considered to depend mainly on the Gs‐mediated pathway. In contrast, chronic hyperglycemia and sustained β‐cell depolarization have been reported to induce a shift from Gs‐dominant to Gq‐dominant signaling, under which GLP‐1 may elicit a more pronounced insulinotropic response through the Gq‐mediated pathway than through the conventional Gs‐mediated pathway, resulting in enhanced insulin secretion through the DAG–PKC pathway and impaired GIP responsiveness (Oduori et al., 2020). The recovery of GIP responsiveness observed in KK‐Ay mice, together with the altered GLP‐1 responsiveness observed in isolated islets, is consistent with the possibility that glucose lowering by luseogliflozin partially corrected the imbalance in G‐protein signaling induced under diabetic conditions (Figure 4).

Reduced GIP responsiveness and its reversibility under diabetic conditions have been reported previously. In diabetic Zucker rats, GIP‐induced insulin secretion, cAMP production, and GIP receptor expression are reduced, which is considered to contribute to GIP resistance (Lynn et al., 2001). In contrast, in ZDF rats, correction of hyperglycemia by phlorizin restored GIP receptor expression and GIP responsiveness (Piteau et al., 2007). In humans with type 2 diabetes, amelioration of blood glucose and DPP‐4 inhibitor treatment have also been reported to improve GIP responsiveness (Aaboe et al., 2015; Højberg et al., 2009). These findings support the concept that impaired GIP responsiveness in diabetes is at least partly reversible and can be restored by improvement of glycemia.

Although luseogliflozin improved blood glucose levels in both KK‐Ay and STZ‐HFD models, the recovery of GIP responsiveness differed markedly between the two models. In KK‐Ay mice, GIP preload after luseogliflozin treatment suppressed the rise in blood glucose after glucose loading, whereas this effect was not observed in STZ‐HFD mice despite improved glycemic control. Notably, insulin secretion experiments using isolated islets from STZ‐HFD mice showed that luseogliflozin treatment altered GLP‐1 responsiveness, suggesting that luseogliflozin may modify incretin responsiveness or intracellular signaling even in residual β‐cells in this model. However, the additional β‐cell injury induced by STZ may have limited the extent to which such cellular changes were reflected as improved glucose tolerance in vivo. In contrast, KK‐Ay mice likely retain sufficient β‐cell mass and secretory capacity for restored GIP responsiveness to translate into a glucose‐lowering effect. Thus, the functional impact of normalized G‐protein signaling and restored GIP responsiveness by SGLT2 inhibition may require a sufficient population of viable and functionally preserved β‐cells.

This study has several limitations. First, because cAMP levels, PKC activation, and G‐protein coupling were not directly measured, the proposed shift from Gq to Gs signaling remains inferential. Second, GIP receptor expression was not directly assessed, and whether luseogliflozin alters Gipr mRNA or GIP receptor protein expression in KK‐Ay and STZ‐HFD islets remains unknown. Third, this study used two mouse models with different degrees of β‐cell dysfunction, and whether similar signaling remodeling occurs in human β‐cells remains to be determined. Fourth, because only male mice were used, it remains unknown whether the effects of luseogliflozin on GIP responsiveness also occur in female diabetic mice. Future studies are needed to directly validate the molecular basis of the transition from Gq to Gs signaling and the recovery of GIP responsiveness, as well as to examine potential sex‐dependent differences in the effects of luseogliflozin. It will also be important to determine whether such signaling remodeling contributes to the durable glycemic improvement and preservation of β‐cell function observed clinically with SGLT2 inhibitors.

## 5 CONCLUSION

Chronic luseogliflozin treatment restored GIP responsiveness in KK‐Ay diabetic mice but not in STZ‐HFD mice, despite improved glycemic control in both models and ex vivo changes in incretin responsiveness in isolated diabetic islets. These findings indicate that SGLT2 inhibition can restore impaired GIP responsiveness under diabetic conditions, but that this effect requires sufficient residual β‐cell function.

## AUTHOR CONTRIBUTIONS

**Quan Yingyue:** Formal analysis; investigation; methodology; visualization. **Harumi Takahashi:** Investigation; methodology. **Norihide Yokoi:** Methodology; resources. **Wataru Ogawa:** Conceptualization; funding acquisition; supervision. **Kenji Sugawara:** Conceptualization; formal analysis; project administration; visualization.

## FUNDING INFORMATION

This work was supported by Taisho Pharmaceutical Co., Ltd. The funder had no role in the study design, data collection, analysis, interpretation, manuscript preparation, or decision to submit the article for publication.

## CONFLICT OF INTEREST STATEMENT

This study was supported by Taisho Pharmaceutical Co., Ltd. The authors declare no other competing interests. The authors had full access to the study data and controlled the decision to submit the manuscript for publication.

## ETHICS STATEMENT

All animal experiments were approved by the Institutional Animal Care and Use Committee of Kobe University Graduate School of Medicine (approval no. 2021‐08‐03) and were conducted in accordance with institutional and national guidelines for the care and use of laboratory animals.

## Data Availability

The data supporting the findings of this study are available from the corresponding author upon reasonable request. *Code Availability Statement*: No custom computer code or algorithm central to the conclusions of this study was used. Data analysis and graph generation were performed using GraphPad Prism 10.
